# The effect of soil on cork quality

**DOI:** 10.3389/fchem.2014.00080

**Published:** 2014-10-13

**Authors:** Miguel N. Pestana, Alberto A. Gomes

**Affiliations:** Instituto Nacional de Investigação Agrária e Veterinária, I.P. Unidade Estratégica de Investigação e Serviços de Agrários e Florestais e Sanidade Vegetal, Quinta do MarquêsOeiras, Portugal

**Keywords:** technology, forest products, soil, numerical taxonomy, cork quality

## Abstract

The present work aimed to contribute for a better knowledge regarding soil features as cork quality indicators for stoppers. Cork sampling was made in eight Cork oak stands (montados de sobreiro) located in the Plio-Plistocene sedimentary formations of Península de Setúbal in southern Tagus River region. The samples used to classify the cork as stopper for wine bottles were obtained in eight cork oak stands, covering soils of different types of sandstones of the Plio-plistocene. In each stand, we randomly chose five circular plots with 30 m radius and five trees per plot with same stripping conditions determined by: dendrometric features (HD- height stipping, PBH- perimeter at breaster height), trees vegetative condition (defoliation degree); stand features (density, percentage canopy cover); site conditions (soil type and orientation). In the center of each plot a pit was open to characterize the soil profile and to classify the soil. Cork quality for stoppers was evaluated according to porosity, pores/per cm^2^ and cork boards thickness. The soil was characterized according to morphological soil profile features (lithology, soil profound, and soil horizons) and chemical soil surface horizon features (organic matter, pH, macro, and micronutrients availability). Based on the variables studied and using the numerical taxonomy, we settled relationships between the cork quality and some soil features. The results indicate: (1) high correlation between the cork caliber and boron, cation exchange capacity, total nitrogen, exchange acidity, and exchangeable magnesium, potassium, calcium, and sodium in soils of theirs cork oaks; (2) the cork porosity is correlated with the number of pores/cm^2^ and magnesium soil content; (3) the other soil features have a lower correlation with the caliber, porosity, and the number of pores per cm^2^.

## Introduction

Cork is the bark of the cork oak tree witch gives rise to cork stoppers used to seal wine bottles. So, the cork is a 100% natural and recyclable product. To produce stoppers with good quality to seal the bottles well, the debarked cork must have specific mechanical features: thickness (caliber) to obtain stoppers with sufficient diameter and low porosity for a good sealing.

In fact cork quality is evaluated differently in the tree, before debarked, or as manufactured stoppers. In the forest, the evaluation is qualitative and subjective, so there is no reproducibility and traceability in its evaluation.

Cork quality is commonly attributed to genetic factors excluding the influence of site conditions (soil and climate). However, it is real that cork features and quality varies widely between different cork-oak regions or even within each region or farm. In any case, it is unknown the influence of the soil features on physical features of the cork. Such knowledge could be very useful to predict cork quality before debarked and its variability on farms.

Thus, given the importance of the cork to the economic sustainability of farms, we think that this article can contribute to a better understanding for the stakeholders regarding the relationships between soil type, soil physical and chemical features, and physical features of cork. This approach is a tool for predicting cork quality on the tree based on the knowledge of the soil.

## Materials and methods

### Material

In this study, 135 trees from 28 circular plots with 30 m radium, distributed by different cork-oak stands at Península de Setúbal, that extends through the region between the Tagus River and the Sado River, were chosen. At least, five trees per plot have been selected for cork samples collection taking in account the uniformity of the soil type. For each plot, the soils were classified according to World Reference Base for soil resources (FAO, 2006).

### Methods

#### Cork sample preparation

Cork samples were obtained by direct extraction form the trees. One sample with 20 × 20 cm square was obtained from each selected tree. Then, the cork samples were boiled for 1 h in order to improve its mechanical capabilities (thickness and porosity), placed in a ventilated area and pressed in order to straighten the corks until to be boards, so that subsequent procedures were more workable.

#### Cork thickness

The thickness of cork boards was measured with a caliper to an accuracy of 0.02 mm. The measurements were performed at both ends of the boards. The thickness was obtained by the average of the two determinations.

#### Cork porosity

The study of cork porosity was made through image analysis with a digital camera with 6 mega Pixels (Pestana, 2003). The samples were sanded to rectify the surface and subjected to a jet of compresses air to clean the surface, so that they can get an image with clear and defined pores.

In addition, it was also determined the number of pores/cm^2^. These two parameters give a good knowledge regarding cork quality.

#### Soils classification

The soil features and classification were obtained from the observation of the soil profile en each plot. To do this, we performed the holes or pits opening to describe the morphological features along the profile and to obtain samples for physical and chemical analysis. The physical and chemical soils analysis was performed according to the analytical methods at Laboratory Rebelo da Silva (LQARS).

The soil of each plot it was classified according to FAO legend (WRB for soil resources, 2006). According to this classification, taking into account the profiles morphological characteristics and the analytical results of the soil, three dominant soil types were identified among the 27 plots: 8 plots are located in Dystric Cambisolos, 10 plots in Albic Arenosols, and 6 plots in Plinthic Podzols.

#### Soils analysis

For the present study the following morphological and physical parameters of soil profile were considered: presence of groundwater horizons, depth to which the presence of cork oak roots was observed, thickness of surface horizon and total thickness of the surface and sub-surface horizons. Regarding the chemical parameters only the values observed in the surface horizon of the soil were considered for the following parameters: organic matter content, total nitrogen and organic carbon, assimilable phosphorus, and potassium (P_2_O_5_, K_2_O), micronutrients (Mn, and B), pH, exchange acidity, and the exchangeable bases (Ca^2+^, Mg^2+^, K^+^, and Na^+^).

The analytical methods used for soil features characterization were:

Organic matter it was calculated multiplying the organic carbon content calculated by the Tinsley method by the factor 1.724. Results were expressed in weight of C per kg of soil.
Nitrogen was determined by catalytic pyrolysis using the auto-analyzer NSC2000. Results are expressed in weight of N per kg of soil of dry matter.
The pH was determined by potentiometry in water using a suspension 1:2.5 of the soil sample.
The extractable phosphorus and potassium were determined by the method of Egner-Riehm modified using an extractant solution of ammonium lactate and acetic acid at pH 3.7–3.8. The phosphorus was quantified by colorimetry according vanamolibdato ammonium method and potassium by flame photometry. Results are expressed mg/kg of P and K per kg of soil (mgkg^−1^).
The extractable Magnesium it was determined ammonium acetate at pH 7 method. The results are expressed in mg of Mg per kg of soil.
Exchangeable bases (Ca^++^, Mg^++^, K^+^, and Na^+^) and exchangeable acidity (H^+^ + Al^3+^) were determined by the Mehlich method (1958) using an extractant solution of barium-triethanolamine chloride adjusted to pH 8.1. Ca and Mg were assayed by atomic absorption spectrophotometry with addition of lanthanum chloride and K and Na by flame photometric. The results are expressed in mg per kg of soil cmol (+). Kg^−1^, and the saturation degree of saturation expressed as a percentage.

Extractable micronutrients (Fe, and B): Fe was determined by Lakanen e Ervio Method (1971) using an extractable solution of ammonium nitrate (5 M) with acetic acid (0.5 M) and EDTA sodic salt (0.02 M) adjusted at pH 4.65. Results were obtained by absorption spectrophotometry and expressed in mg of each micronutrient per kg of soil; Boron was extracted in boiling water for 5 min and titrated by Curcumin-oxalic acid method (LQARS, 1988). The results are expressed in mg of B per kg of soil.

### Data statistical analysis

The plots characterization was supported by the average values of physical parameters of cork. This procedure is due to the fact that it is impossible to accurately determine the soil parameters for each tree, thus obtaining the mean values of these cork features. In Table 1, we have adopted codes for the features studied.

**Table 1:** **Correspondence between the variables and the codes adopted**.

**Features**	**Units**	**Code**
Caliber	(mm)	Caliber
Porosity	(%)	Poro
Number of pores per cm^2^		Npor
Surface horizon thickness	(cm)	EspA
Total surface and subsurface horizons thickness	(cm)	EspA+B
Organic matter content	(gkg^−1^)	MO
Exchangeable calcium	(cmol_(c)_ kg^−1^)	Ca^++^
pH		pH
Exchangeable magnesium	(cmol_(c)_ kg^−1^)	Mg^++^
Exchangeable potassium	(cmol_(c)_ kg^−1^)	K^+^
Exchangeable sodium	(cmol_(c)_ kg^−1^)	Na^+^
Cation exchange capacity	(cmol_(c)_ kg^−1^)	CTC
Base saturation degree	(cmol_(c)_ kg^−1^)	GSB
Exchange acidity	(cmol_(c)_ kg^−1^)	AT
Exchangeable bases sum	(cmol_(c)_ kg^−1^)	S
Total nitrogen	(gkg^−1^)	Ntot
Extratable phosphorus	(mgkg^−1^)	P2O5
Extratable potassium	(mgkg^−1^)	K2O
Extratable magnesium	(mgkg^−1^)	Mg
Boron	(mgkg^−1^)	B

#### Numerical taxonomy analysis

For each data sets was prepared (in advance) a data matrix were rows correspond to the plots Operational Taxonomic Units (OTUs), and the columns correspond to the variables determined (Pestana, 2003).

How there is a different nature of the various variables, we proceeded to the standardization of the original matrix. Then, we obtain a new matrix with standardized data, where the average value of each feature is now zero and its variance 1. In this operation, it is calculated the mean, and standard deviation for each feature and then it replaces each original value by dividing their difference to the mean and its standard deviation (Carneiro, 1987; Pestana, 2003).

We calculated the similarity coefficient using the average Euclidean distance. This coefficient represents the distance between the representative points of two samples in space, which will have as many dimensions as the number of features used. In case of equal analyzing objects, this distance is zero and increases with the dissimilarity between then (Carneiro, 1987; Pestana, 2003).

Of the various methods of aggregation of sequential, agglomerative, hierarchical and non-override type, i.e., the type SAHN, we used the Unweighted Pair—Group Method Using Arithmetic Averages (UPGMA) (Sneath and Sokal, 1973; Pestana, 2003).

The results thus obtained are presented on the form of a branched structure, in which the different branches are related according to the values of the similarity measures which are based on the agglomeration method, which is known as phenogram (Pestana, 2003).

For this phenogram, we calculated the cophenetic correlation coefficient (Sokal and Rohlf, 1962) between the matrix of cophenetic values, expressing the relationship of similarity between the implicit OTUs in phenogram, and similarity matrix (or dissimilarity) was calculated. The cophenetic correlation coefficient indicates the degree of agreement between the two matrixes, allowing assess whether the phenogram is an acceptable representation of those distances (Carneiro, 1987; Pestana, 2003).

It used also another method of aggregation for better understanding of the results the aggregation method called Minimum Spanning Tree (MST). To obtain the graphical representation of the studying objects along axes, a small number of dimensions, usually two or three, retaining as much of the variability of the original multidimensional matrix data, we selected the ordination method on main components. The projections of the variables that characterize the objects studied in the first two main components, which allows us to analyze the contribution of each of the spatial arrangement of the objects studied were made (Reis, 2001).

## Results and discussion

To perform this analysis, we used a data matrix with 28 plots (rows) and the 20 physical features of corks (including image analysis) and physical and chemical soil features (columns, Table 2).

**Table 2:** **Cork quality and physical and chemical soil data**.

**Plots**	**Cork quality data**			**Soil physical data**			**Soil chemical data**													
	**Poro**	**Npor**	**Caliber**	**EspA**	**EspA+B**	**MO**	**Ntot**	**pH**	**P_2_O_5_**	**K_2_O**	**Mg**	**B**	**Ca^++^**	**Mg^++^**	**K^+^**	**Na^+^**	**AT**	**S**	**CTC**	**GSB**
CLMPS/2	18.91	0.06	28.63	15.0	40.0	1.78	0.06	5.7	53.0	49.0	25.0	0.05	1.46	0.25	0.09	0.11	1.50	1.92	3.4	56.2
CLMPS/3	20.07	0.06	34.93	17.5	32.0	1.05	0.03	5.8	12.0	29.0	13.0	0.05	0.77	0.15	0.06	0.02	1.00	0.99	2.0	49.9
CLMPS/4	38.80	0.07	40.53	15.0	22.5	0.23	0.01	5.7	3.0	37.0	9.0	0.05	0.30	0.07	0.07	0.03	1.00	0.46	1.5	31.7
CLMPS/5	32.33	0.08	33.76	15.0	35.0	1.91	0.06	5.6	33.0	124.0	18.0	0.05	1.35	0.17	0.15	0.02	1.50	1.70	3.2	53.1
CLMM/2	12.87	0.05	30.91	15.0	40.0	1.34	0.04	5.7	12.0	47.0	19.0	0.05	1.26	0.17	0.09	0.02	1.40	1.54	2.9	52.3
CLMM/3	19.26	0.07	31.02	12.5	32.5	1.24	0.04	6.1	17.0	49.0	23.0	0.05	0.96	0.21	0.10	0.04	0.70	1.31	2.0	65.2
CLMM/4	30.66	0.05	29.19	20.0	42.5	0.91	0.03	5.8	9.0	25.0	14.0	0.05	0.81	0.15	0.05	0.12	1.00	1.12	2.1	52.8
CLMM/5	31.24	0.06	32.01	12.5	30.0	1.08	0.03	5.6	6.0	32.0	12.0	0.05	0.85	0.13	0.06	0.12	1.90	1.16	3.1	38.0
HEMO5	3.97	3.68	37.69	22.5	60.0	0.88	0.03	8.3	69.0	40.0	44.0	0.05	4.47	0.42	0.05	0.03	0.00	4.97	5.0	100.0
HEMO6	3.97	3.07	34.01	30.0	72.5	0.86	0.03	5.4	4.0	151.0	232.0	0.58	1.50	2.27	0.37	0.08	2.00	4.22	6.2	67.8
HEMR14	5.20	3.83	35.67	27.5	47.5	0.40	0.01	5.4	9.0	39.0	19.0	0.05	1.25	0.17	0.08	0.01	1.70	1.51	3.2	47.0
HEMR14a	5.30	5.12	31.51	20.0	35.0	2.13	0.06	6.5	17.0	101.0	30.0	0.33	1.93	0.32	0.22	0.03	1.00	2.49	3.5	71.4
HEPMR42	13.21	7.18	36.25	25.0	47.5	1.24	0.03	7.4	28.0	68.0	42.0	0.05	2.51	0.29	0.11	0.02	0.00	2.94	2.9	100.0
HEPMR42a	4.21	4.71	25.45	20.0	50.0	1.27	0.04	7.2	27.0	48.0	43.0	0.25	2.55	0.44	0.09	0.02	0.00	3.10	3.1	100.0
HEPMR44	4.04	3.29	31.94	20.0	47.5	0.92	0.02	6.2	9.0	39.0	25.0	0.05	0.89	0.23	0.08	0.01	1.10	1.21	2.3	52.4
HEPMR44a	8.23	5.82	36.45	20.0	45.0	1.38	0.04	7.4	25.0	36.0	13.0	0.05	2.35	0.28	0.06	0.02	0.00	2.71	2.7	100.0
HEPMR52a	3.71	0.03	29.29	30.0	60.0	1.09	0.02	6.2	7.0	36.0	11.0	0.05	0.83	0.24	0.08	0.02	1.20	1.17	2.4	49.3
VCVC1	8.99	5.42	34.46	15.0	45.0	1.31	0.04	5.7	15.0	95.0	18.0	0.05	0.68	0.19	0.26	0.01	1.90	1.13	3.0	37.4
VCVC2	6.54	4.99	40.67	22.5	27.5	0.80	0.02	5.3	13.0	21.0	13.0	0.05	0.48	0.11	0.05	0.01	1.50	0.64	2.1	30.0
VCVC3	6.71	3.85	42.92	17.0	21.0	0.98	0.03	5.3	14.0	41.0	46.0	0.05	0.80	0.17	0.07	0.03	1.60	1.06	2.7	39.9
VCVC4	8.17	4.61	50.52	17.5	35.0	2.54	0.07	5.9	12.0	119.0	90.0	0.05	2.00	0.80	0.23	0.04	1.70	3.07	4.8	64.4
VCVC5	7.61	4.61	46.96	20.0	35.0	1.90	0.06	6.0	15.0	102.0	70.0	0.26	1.59	0.43	0.21	0.02	1.30	2.25	3.5	63.4
VC1	4.01	3.61	32.32	30.0	45.0	1.60	0.04	5.7	5.0	35.0	40.0	0.19	0.98	0.25	0.10	0.02	1.60	1.35	3.0	45.7
VC2	2.48	3.53	30.26	25.0	65.0	1.30	0.03	5.2	8.0	24.0	36.0	0.19	0.78	0.20	0.05	0.01	1.70	1.05	2.8	38.2
VC3	5.69	3.71	26.17	25.0	80.0	0.08	0.03	5.5	5.0	24.0	40.0	0.19	0.60	0.19	0.05	0.01	1.30	0.85	2.2	39.6
VC4	3.87	3.10	31.00	30.0	50.0	1.20	0.04	5.4	7.0	31.0	24.0	0.19	0.71	0.19	0.06	0.02	1.90	0.98	2.9	33.9
VC5	5.26	3.86	29.25	25.0	70.0	1.55	0.05	5.4	10.0	41.0	44.0	0.19	0.96	0.30	0.09	0.01	1.30	1.36	2.7	51.0
CI	2.84	1.90	21.00	25.00	65.00	0.85	0.03	5.4	5.0	20.0	24.0	0.19	0.35	0.12	0.05	0.01	1.90	0.53	2.4	21.7

**Figure 1:**
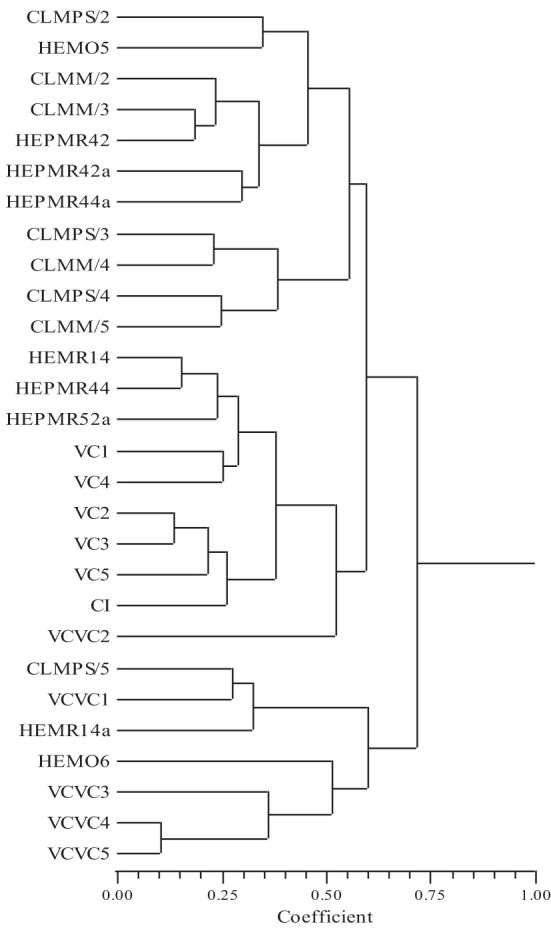
**Phenogram of 28 plots, based on the UPGMA method applied to the distances matrix (*r* = 0.72)**.

The phenogram (Figure 1) obtained from the distance matrix (using the UPGMA method) is suitable for the respective matrix because of the cophenetic coefficient correlation (*r* = 0.72). Observing the phenogram it is possible to verify the establishment of two groups. One made by plots CLMPS/5, VCVC1, HEMR14a, HEMO6, VCVC3, VCVC4, and VCVC5 and another with the remaining plots. This last group, we might consider subdivide into two groups composed by: the first includes the CLMPS/2, HEMO5, CLMM2, CLMM3, HEPMR42, HEPMR42a, HEPMR44a, CLPMS/3, CLMM4, CLPMS4, and CLMM/5; the second group is composed by the remaining plots.

**Figure 2:**
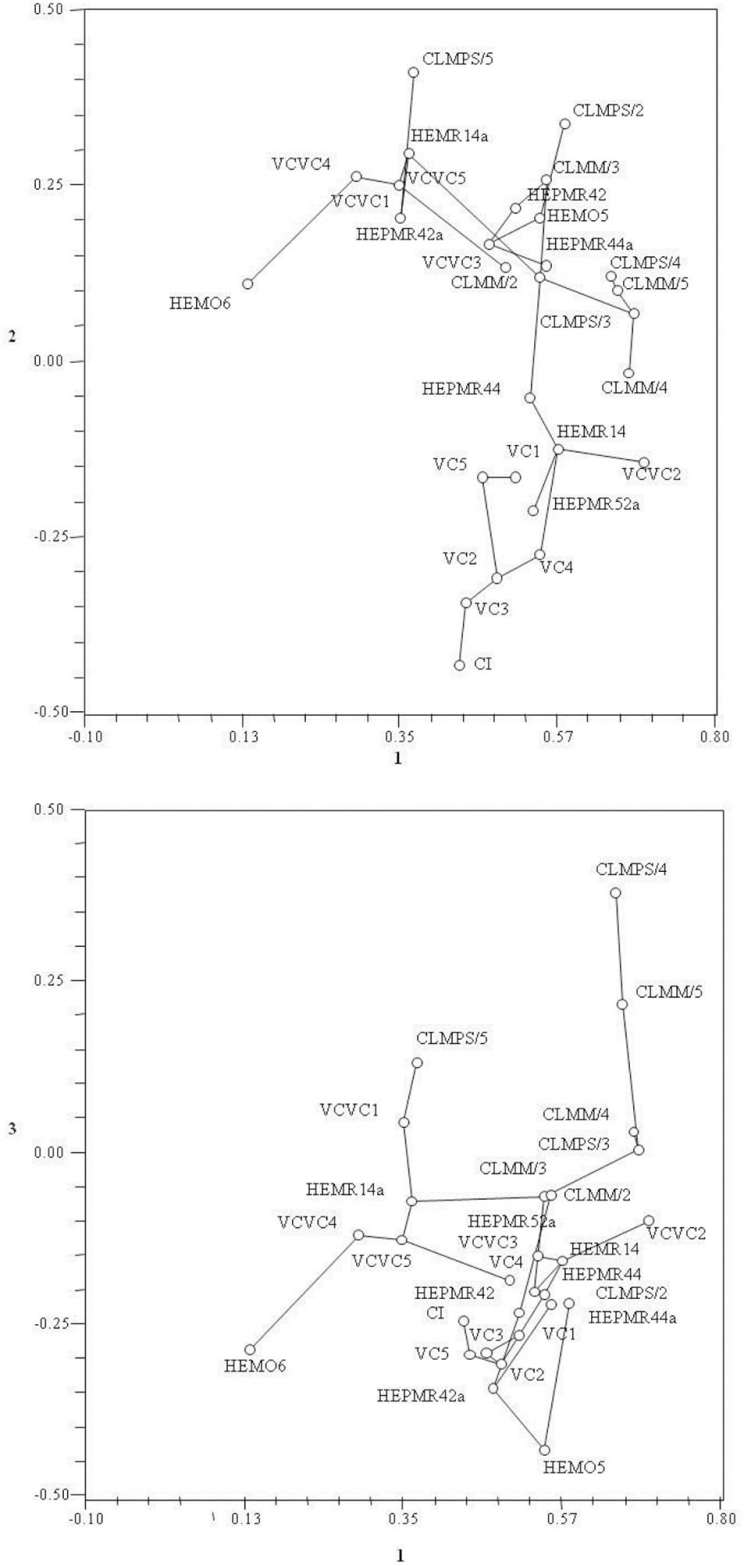
**Projections of the 28 plots in the plane defined by the two first main axes, which was overlying the Minimum Spanning Tree**.

The projection of the 28 plots in the spatial defined by the three main axes, which together account for 77.71% of total variance, which was overlaid with MST (Figure 2), allows to confirm the groupings obtained by the phenogram. The analysis of Figure 2 allows us to say that the links and the spatial arrangement of the plots are in agreement with most clusters determined in the phenogram.

**Figure 3:**
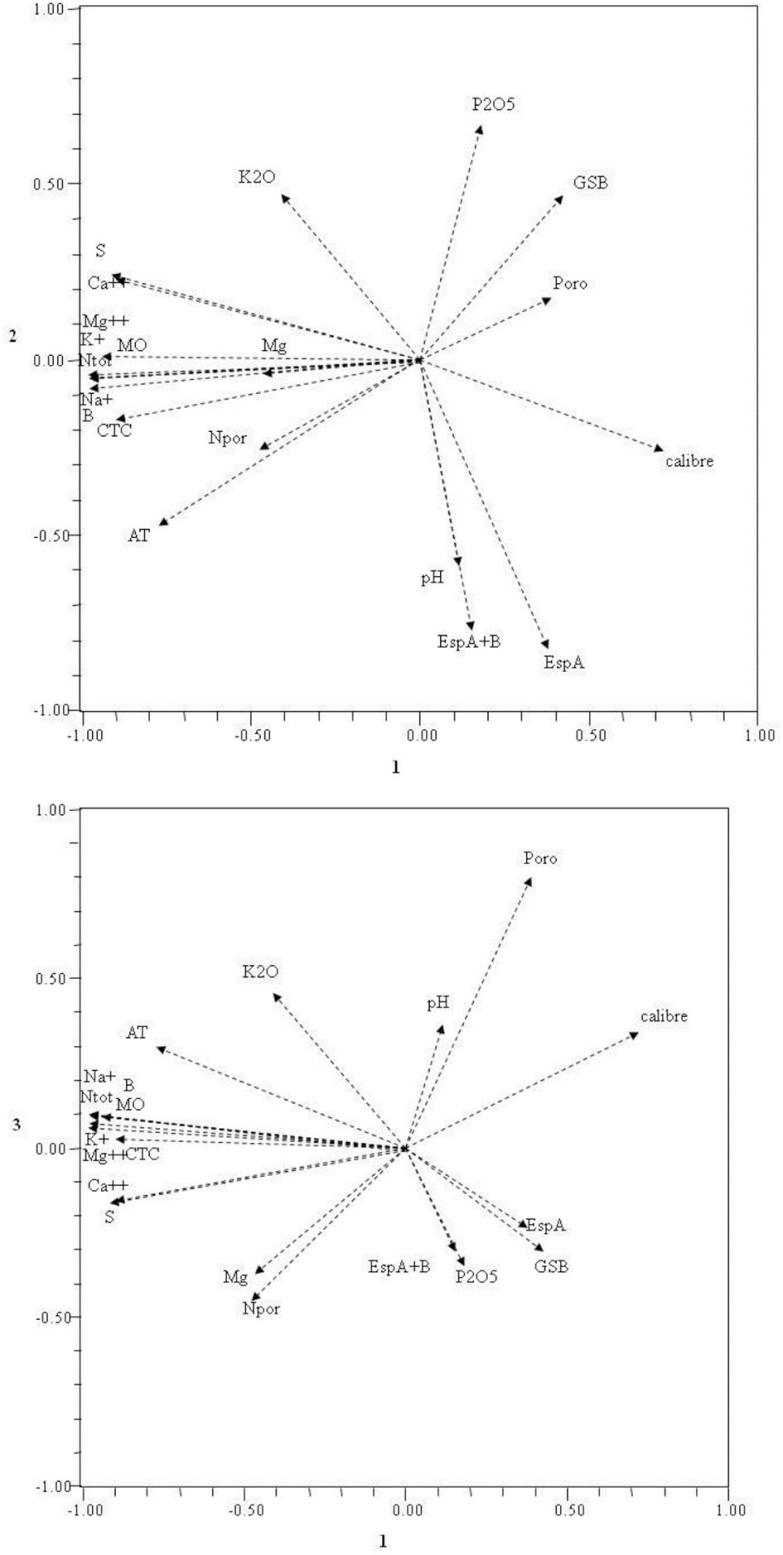
**Projections of the physical and chemical soil features, image analysis, and physical cork features, in the first two main components**.

Figure 3 shows the contributions of variables to the spatial distribution of the plots.

The first main component is controlled by organic matter (MO), total nitrogen (Ntot), boron (B), exchangeable calcium (Ca^++^), magnesium (Mg^++^), potassium (K^+^), and sodium (Na^+^), exchangeable bases sum (S), caption exchange capacity (CTC), exchange acidity (AT), and caliber (Caliber). The second component is controlled by the most important variables for pH (pH), surface horizon thickness (EspA), total surface and subsurface horizons thickness (EspA+B), phosphates (P2O5), potassium oxide (K2O), and sum of basic captions (GSB). The third is controlled by the magnesium (Mg), porosity (Pore), and pores number/cm^2^ (Npor).

Analyzing the two Figures 2, 3, we find that the first main component separates to the right side, the plots with differences in cork caliber, and to the left, the soil features in total nitrogen, caption exchange capacity, organic matter, boron, sulfur, and exchangeable sodium, magnesium, calcium, and potassium.

The second main component drives to the down region of the same figures (Figures 2, 3), plots with high values of surface horizon thickness, total surface and sub-surface horizons thickness and pH, and to upper region the potassium oxide, phosphates, and sum of basic captions. The third main component drives the plots with corks high porosity to the upper side of the plane (1,2) and the magnesium and number of pores/cm^2^ to the opposite side of the same plane.

We see also a high correlation between the caption exchange capacity and the exchangeable calcium, organic matter, total nitrogen, exchangeable magnesium, potassium, and sodium that indicates that these features are mutually dependents.

We found that the plots of corks with high porosity and low number of pores/cm^2^ are from corks on soils containing lower levels of magnesium. Lower values of boron, cation exchange capacity, total nitrogen, exchange acidity, and exchangeable magnesium, potassium, calcium, and sodium are in plots where de corks have a high caliber.

## Conclusions

Regarding to the foregoing, we conclude that there is a high correlation between the cork caliber and boron, caption exchange capacity, total nitrogen, the exchange acidity, and exchangeable magnesium, potassium, calcium, and sodium in soils of theirs cork oaks, i.e., lower values of boron, cation exchange capacity, total nitrogen, the exchange acidity, and exchangeable magnesium, potassium, calcium, and sodium are in plots where de corks have a high caliber. It was also found that the cork porosity is correlated with the number of pores/cm^2^ and magnesium, i.e., the number of pores/cm^2^ and magnesium varies inversely with the cork porosity.

The other soil features have a lower correlation with the caliber, porosity, and the number of pores per cm^2^.

### Conflict of interest statement

The authors declare that the research was conducted in the absence of any commercial or financial relationships that could be construed as a potential conflict of interest.
